# Effects of long-acting muscarinic antagonists on promoting ciliary function in airway epithelium

**DOI:** 10.1186/s12890-022-01983-3

**Published:** 2022-05-08

**Authors:** Mineo Katsumata, Tomoyuki Fujisawa, Yosuke Kamiya, Yuko Tanaka, Chiaki Kamiya, Yusuke Inoue, Hironao Hozumi, Masato Karayama, Yuzo Suzuki, Kazuki Furuhashi, Noriyuki Enomoto, Yutaro Nakamura, Naoki Inui, Masato Maekawa, Mitsutoshi Setou, Hiroshi Watanabe, Koji Ikegami, Takafumi Suda

**Affiliations:** 1 Second Division, Department of Internal Medicine, Hamamatsu University School of Medicine, 1-20-1 Handayama, Higashi-ku, Hamamatsu, Shizuoka 431-3192 Japan; 2 Department of Clinical Pharmacology and Therapeutics, Hamamatsu University School of Medicine, 1-20-1 Handayama, Higashi-ku, Hamamatsu, 431-3192 Japan; 3 Department of Laboratory Medicine, Hamamatsu University School of Medicine, 1-20-1 Handayama, Higashi-ku, Hamamatsu, Shizuoka 431-3192 Japan; 4 Department of Cellular and Molecular Anatomy and International Mass Imaging Center, Hamamatsu University School of Medicine, 1-20-1 Handayama, Higashi-ku, Hamamatsu, 431-3192 Japan; 5 Department of Anatomy and Developmental Biology, Graduate School of Biomedical and Health Sciences, Hiroshima University, 1-2-3 Kasumi, Minamiku, Hiroshima, 734-8553 Japan

**Keywords:** Asthma, Airway epithelium, Ciliary beat frequency, COPD, Long-acting muscarinic antagonists, Mucociliary clearance

## Abstract

**Background:**

Mucociliary clearance (MCC) is an essential defense mechanism in airway epithelia for removing pathogens from the respiratory tract. Impaired ciliary functions and MCC have been demonstrated in asthma and chronic obstructive pulmonary disease (COPD). Long-acting muscarinic antagonists (LAMAs) are a major class of inhaled bronchodilators, which are used for treating asthma and COPD; however, the effects of LAMAs on ciliary function remain unclear. This study aimed to identify the effects of LAMAs on airway ciliary functions.

**Methods:**

Wild-type BALB/c mice were treated with daily intranasal administrations of glycopyrronium for 7 days, and tracheal samples were collected. Cilia-driven flow and ciliary activity, including ciliary beat frequency (CBF), ciliary beating amplitude, effective stroke velocity, recovery stroke velocity and the ratio of effective stroke velocity to recovery stroke velocity, were analyzed by imaging techniques. Using in vitro murine models, tracheal tissues were transiently cultured in media with/without LAMAs, glycopyrronium or tiotropium, for 60 min. Cilia-driven flow and ciliary activity were then analyzed. Well-differentiated normal human bronchial epithelial (NHBE) cells were treated with glycopyrronium, tiotropium, or vehicle for 60 min, and CBF was evaluated. Several mechanistic analyses were performed.

**Results:**

Intranasal glycopyrronium administration for 7 days significantly increased cilia-driven flow and ciliary activity in murine airway epithelium. In the murine tracheal organ culture models, treatment with glycopyrronium or tiotropium for 60 min significantly increased cilia-driven flow and ciliary activity in airway epithelium. Further, we confirmed that 60-min treatment with glycopyrronium or tiotropium directly increased CBF in well-differentiated NHBE cells. In the mechanistic analyses, neither treatment with glycopyrronium nor tiotropium affected intracellular calcium ion concentrations in well-differentiated NHBE cells. Glycopyrronium did not increase protein kinase A activity in well-differentiated NHBE cells. Moreover, glycopyrronium had no effect on extracellular adenosine triphosphate concentration.

**Conclusions:**

LAMAs exert a direct effect on airway epithelium to enhance ciliary function, which may improve impaired MCC in asthma and COPD. Further investigations are warranted to elucidate the underlying mechanisms of the effects of LAMAs on the promotion of airway ciliary function.

**Supplementary Information:**

The online version contains supplementary material available at 10.1186/s12890-022-01983-3.

## Background

The airway epithelium plays a pivotal role in the host’s defense against inhaled foreign substances such as viruses, bacteria, and airway pollutants, serving as a physical barrier and regulator of innate and adaptive immune responses [1–5]. Mucociliary clearance (MCC) is an innate frontline defense mechanism in the airway epithelium that removes inhaled pathogens from the airways [6, 7]. The functional components of MCC are the protective mucus layer, airway surface liquid layer, and cilia on the ciliated cells, which comprise a major fraction of airway epithelial cells. Adequate ciliary functions are essential for effective MCC in transporting foreign materials, such as viruses and bacteria, trapped in the mucous layer towards the mouth. Ciliary beat frequency (CBF) is a major determinant of ciliary function [8–10]. The importance of ciliary function in the host’s airway defense is well documented in primary ciliary dyskinesia, in which genetic defects in cilia structure and motility impair MCC and are associated with recurrent airway infection [11]. We recently showed that influenza A virus infection in the airway epithelium stimulated ciliary function, which hastened the elimination of viruses from the respiratory tract as one of the host’s initial defense responses [10].

Asthma and chronic obstructive pulmonary disease (COPD) are chronic airway diseases characterized by symptomatic airflow limitation [12–14], and both are known to have impaired ciliary function and MCC. Studies have demonstrated that CBF was decreased in patients with moderate to severe asthma and those with asthma with sputum neutrophilia [15, 16]. In patients with COPD, CBF reductions in ciliated cells were observed especially in moderate to severe COPD [17, 18]. Ciliary dysfunction in asthma and COPD causes impairment of mucus clearance, resulting in worsening of airway obstruction and susceptibility to infection. Restoring ciliary function is therefore an important therapeutic target for patients with asthma and COPD. inhaled corticosteroids (ICS), long-acting β2-agonists (LABAs), and long-acting muscarinic antagonists (LAMAs) have been widely used in real-world clinical practice to treat asthma and COPD [13, 14]. LABAs and LAMAs act as bronchodilators in patients with asthma and COPD, and β2 agonists have been shown to modulate ciliary function and MCC [17, 19, 20]. A study demonstrated that salmeterol, one of the major LABAs, increased CBF in nasal epithelial cells [17].

In contrast to LABAs, there is scarce evidence for the effects of LAMAs on ciliary function in airway epithelium. LAMAs, such as tiotropium bromide (TIO), glycopyrronium bromide (GLY), and aclidinium bromide, inhibit the muscarinic receptors of the bronchioles and relax airway smooth muscle. The M3 receptor, a muscarinic receptor subtype, is highly expressed in smooth muscles cells, submucosal glands, and airway epithelial cells in the lungs [21, 22]. Given that LAMAs have strong selectivity for the M3 receptor, LAMAs might act on airway epithelium, and have modulatory actions on ciliary functions in the airway epithelium.

In the present study, we investigated the effects of LAMAs on airway ciliary functions using in-vivo mouse models, organ cultures of murine tracheal tissue, and primary culture of normal human bronchial epithelial (NHBE) cells. Here, we demonstrate that LAMAs have a direct effect on airway epithelium to enhance ciliary function to improve MCC.

## Methods

### Mice

BALB/c mice were purchased from Japan SLC, Inc. (Shizuoka, Japan), and all experiments involving the mice followed protocols approved by the Animal Care and Use Committees of Hamamatsu University School of Medicine, Shizuoka, Japan (license number H31-047) and were performed in accordance with the relevant guidelines and regulations.

### Chemicals

GLY and TIO were purchased from Sigma-Aldrich (St. Louis, MO, USA) and dissolved in phosphate-buffered saline (PBS). GLY was used at 300µM for the intranasal administration to mice and at 100 nM for the organ culture of murine tracheal tissue and primary NHBE cell culture under air–liquid interface (ALI) conditions. TIO was used at 100 nM for culture conditions. 2-Aminoethoxydiphenylborane (2-APB), an inhibitor of inositol-trisphosphate receptor, was purchased from Tocris Bioscience (Bristol, UK) and used at 40 μM. N-[2-(p-bromocinnamylamino) ethyl]-5-isoquinolinesulfonamide, dihydrochloride (H89), a protein kinase inhibitor with a high specificity for protein kinase A (PKA), was purchased from Cayman Chemical (Ann Arbor, MI, USA) and used at 10 μM. Telenzepine, M1 receptor antagonist, was purchased from Tocris Bioscience (Bristol, UK) and used at 10 μM. Gallamine, M2 receptor antagonist, was purchased from Sgma-Aldrich and used at 30 μM. 4-DAMP, M3 receptor antagonist, was purchased from Abcam (Cambridge, UK) and used at 10 μM. Thapsigargin were purchased from Sigma-Aldrich and used at 1 μM.

### Isolation of murine trachea and organ culture of tracheal tissue

Tracheas were taken from 9 to 11-week-old female BALB/c mice and placed in cold collection medium solution (Dulbecco's Modified Eagle Medium with sodium pyruvate solution) kept in an ice bath. Excess fat and connective tissue debris were immediately removed from the trachea using forceps. The membranous portion of the trachea was then excised to expose the ciliated epithelium in the trachea. After the dissection, the tracheal tissue was cultured in 2 mL of culture medium in the presence or absence of GLY or TIO in 35-mm culture dishes at 37 °C for 60 min as an organ culture. The tracheal epithelium was then observed and analyzed at room temperature (23–28 °C) using a dedicated microscope.

### Daily intranasal administration of glycopyrronium to mice

The 8-week-old female BALB/c mice were anesthetized with a 3% isoflurane oxygen mixture and administered 25 µL of GLY solution (300 µM) or PBS alone intranasally once daily for 7 days. Mice were sacrificed on day 8, after which tracheas were taken. After the organ culture of trachea was prepared as described above, the tracheal epithelium was immediately observed and analyzed at room temperature (23–28 °C) using a dedicated microscope.

### Cell culture

Primary NHBE cells were purchased from Lonza (catalog no. CC-2541; Basel, Switzerland). NHBE cells in a submerged condition were cultured on 6.5-mm Transwell (Corning, NT, USA) using commercially available bronchial epithelial growth medium (BEGM; Lonza) and incubated at 37 °C in a humidified atmosphere with 5% carbon dioxide. When the NHBE cells reached full confluency in an immersed culture condition, the cells were transferred to an ALI culture condition using ALI culture medium (HBTEC Air–Liquid Interface Differentiation Medium; Lifeline Cell Technology, Frederick, MD, USA), as previously described with slight modifications [3, 4, 23, 24]. The cells were then cultured for 4 weeks in ALI conditions to facilitate polarization and ciliary differentiation for subsequent experiments [25, 26].

### Analysis of cilia-driven flow

Ciliary transport on the surface of the intact murine trachea was analyzed after transient organ culture, as previously described [10]. To visualize the cilia-driven flow, tracheal tissue was placed in a 35-mm culture dish with the luminal face down in 2 mL of culture medium containing 0.2-μm-diameter polystyrene beads (0.2-μm red fluorescent beads: Thermo Fisher Scientific, Waltham, MA, USA). Methylcellulose (M0512, Sigma-Aldrich, St. Louis, MO, USA) was added to the culture medium at a concentration of 0.5% to stabilize the movement of fluorescent beads by increasing the viscosity of the medium. The movement of the beads under the tracheal epithelium was observed from the bottom of the dish using an inverted fluorescence microscope (Eclipse TE2000-U, Nikon, Tokyo, Japan) equipped with a CFI Plan Fluor objective lens (Nikon) and a CCD camera (Hamamatsu Photonics, Hamamatsu, Japan). The optimal configuration of the lens for tracking the movement of the beads, which were 0.5–2 mm from the chamber bottom, was × 20 magnification, an NA of 0.5, and a long working distance (2.1 mm). The velocity of each bead was calculated by dividing the width of the field of view by the time each individual bead took to travel across the field. The fluid movement velocity (cilia-driven flow) was calculated using the beads’ migration distance and travel time by tracking individual fluorescent beads using Aquacosmos image analysis software (Hamamatsu Photonics). Three or more independent tracheal samples in each condition were analyzed. In each tracheal sample, 10 fields of view and 5 beads in each field were evaluated, which meant that cilia-driven flows of 50 beads in one trachea were analyzed. In each experiment, mean data were calculated by determining the average of cilia-driven flows for each trachea, and then averaging data for all tracheae in each condition. The rainbow trace was depicted using a macro for the free software, ImageJ, from Hiratsuka laboratory of JAIST (https://www.jaist.ac.jp/ms/labs/hiratsuka/, in Japanese only).

### Analysis of the ciliary beating orientation

The analysis was performed as described previously [8–10] with slight modifications. After the murine tracheal tissue was transiently cultured in culture medium, the cilia tips of the ciliated cells were labeled with Indian ink diluted with culture medium (1:100) to analyze the intact cilia. The motility of the ink-labeled cilia tips was recorded using HAS-L1 and HAS-U1 high-speed cameras (DITECT Co. Ltd, Tokyo, Japan) at 300 fps to reflect ciliary motility. The recording was performed at 23–28 °C. CBF was determined by subjecting the original traces to a fast Fourier transform using Microsoft Excel. The ciliary beating amplitude, effective stroke velocity, recovery stroke velocity, and the ratio of effective stroke velocity to recovery stroke velocity were calculated from the movement of the cilia tips. The CBF data are presented as the median (ranges). The ciliary beating amplitude, effective stroke velocity, recovery stroke velocity, and effective stroke velocity-to-recovery stroke velocity ratio are presented as the mean ± SEM. Fully differentiated NHBE cells cultured under ALI conditions for 4 weeks were also used to measure CBF. Three independent tracheal samples and 10 ink-labeled cilia in each sample were analyzed (n = 30 ink-labeled cilia for each condition). Kymographs of ciliary beating were depicted with a macro embedded in ImageJ.

### Monitoring of intracellular calcium ion concentrations

Intracellular calcium ion (Ca^2+^) concentrations were measured using fura-2/AM, a fluorescent Ca^2+^ indicator. NHBE cells cultured under ALI conditions for 4 weeks were incubated with 2.5 μM fura 2-AM (Dojindo, Kumamoto, Japan) for 30 min at 37 °C, and the cells were treated with GLY or TIO at each concentration. Fluorescent images of fura-2/AM were acquired and quantified every 30 s or 120 s from individual cells with a fluorescence analyzer (Aquacosmos, Hamamatsu Photonics, Hamamatsu, Japan) using an ultra-high sensitivity camera. Changes in the fluorescence ratio (F340/F380) of fura-2 were used to express changes in the intracellular Ca^2+^ concentrations. Thapsigargin (TG), an inhibitor of the endoplasmic reticulum Ca^2+^-ATPase pump, which causes a transient increase of intracellular Ca^2+^ concentrations [27], was used for positive controls.

### Analysis of protein kinase A (PKA) activity

NHBE cells cultured under ALI conditions for 4 weeks were treated with GLY for 60 min. The cells were then harvested using RIPA buffer (ATTO, Tokyo, Japan) and PKA activity was determined using nonradioactive PKA activity assay kits (Enzo Life Sciences, Ann Arbor, MI, USA), according to the manufacturer’s instructions. Briefly, the PKA substrate microtiter plates were soaked in kinase assay dilution buffer at room temperature. The cell lysates (1 μg of protein) were subsequently added, followed by the addition of adenosine triphosphate (ATP) to initiate the reaction. After incubation at 30 °C for 90 min, the phosphor-specific substrate antibody was added, and the reaction was incubated at room temperature for 1 h. The HRP-conjugated secondary anti-rabbit IgG was subsequently added to each well, and incubated for an additional 30 min. The TMB substrate solution was added to each well, and further incubated for 30 min. Lastly, the stop solution was added, and the 96-well plate was read at 450 nm in a microplate reader.

### Adenosine triphosphate (ATP) measurements

ATP concentrations were measured in culture supernatants using an ATP assay kit based on luminometric techniques (Lucifell 250 plus, Kikkoman Biochemifa, Tokyo, Japan) according to the manufacturer’s protocol. In total, 100 µL of culture medium from murine tracheal tissue with or without GLY were used. Briefly, 100 µL of the ATP extraction reagent was added to each sample, and after 20 s, luciferin-luciferase (100 µL) was added to each sample. The luminescence of each sample was measured using a Lumitester C-100 (Kikkoman Biochemifa).

### Statistical analysis

All statistical analyses were performed with EZR (Saitama Medical Center, Jichi Medical University), which is a graphical user interface for R software (version 2.4–0, The R Foundation for Statistical Computing, Vienna, Austria). A student’s t-test or Tukey’s test were used to compare means among groups. Distribution of CBF data deviated from normal distribution, and were analyzed by the Mann–Whitney U-test or Kruskal–Wallis test based on the number of groups. All data, except for CBF, are presented as means ± SEM. The CBF data are presented as the median (range). Statistical significance was assigned when *p* values were ≤ 0.05.

## Results

### Continuous intranasal administration of GLY promotes cilia-driven flow and ciliary activity in the murine tracheal epithelium

**Fig. 1:**
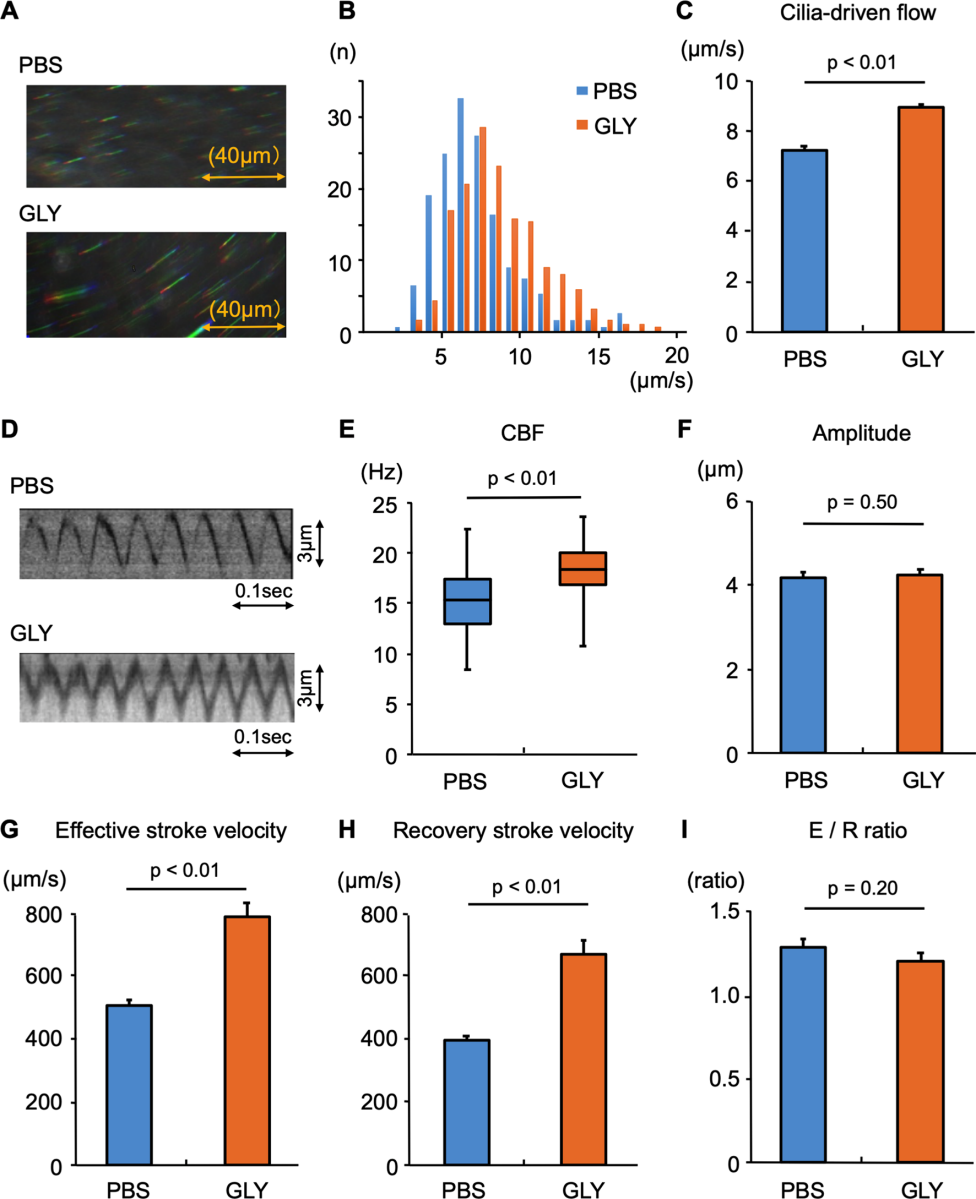
The effects of the intranasal administration of glycopyrronium on cilia-driven flow and ciliary motility Wild-type (WT) mice were treated with glycopyrronium (GLY) or phosphate-buffered saline (PBS) intranasally for 7 days, The trachea was then removed. Cilia-driven flow and ciliary motility were evaluated. **A.** Representative bead trajectories of cilia-driven flow (rainbow trace for 4.4 s). **B.** the histogram of the cilia-driven flows (150 beads from 3 tracheas) in each condition. **C.** the bar chart of the cilia-driven flow demonstrated that 7-day GLY administration significantly increased cilia-driven flow (PBS, 7.30 ± 0.15 μm/s; GLY, 9.04 ± 0.08 μm/s; n = 3 tracheas in each condition). **D.** Kymographs of the ciliary beating. **E.** GLY significantly increased ciliary beating frequency (CBF; PBS, 15.23 [8.20–22.27] Hz; GLY, 18.17 [10.55–23.44] Hz; n = 30 cilia in each condition). **F.** The ciliary beating amplitude did not differ between the conditions (PBS, 4.18 ± 0.14 μm; GLY, 4.23 ± 0.15 μm). **G** and **H.** The effective stroke velocity (**G**) (PBS, 503.7 ± 21.1 μm/s; GLY, 785.7 ± 45.1 μm/s) and recovery stroke velocity (**H**) (PBS, 396.8 ± 15.3 μm/s; GLY, 675.3 ± 43.3 μm/s) were significantly increased by GLY administration compared with the control. **I.** The ratio of effective stroke velocity to recovery stroke velocity (E/R ratio) did not differ between the two conditions (PBS, 1.29 ± 0.05; GLY, 1.20 ± 0.05)

We initially investigated whether the daily administration of GLY promotes ciliary functions in the murine tracheal epithelium. Wild-type (WT) mice were treated with GLY or PBS intranasally for 7 days. After 7 days of treatment, we resected the tracheal samples and analyzed the cilia-driven flow over the tracheal surface using small polystyrene beads in each condition. Representative bead trajectories and movies are presented in Fig. 1A and Additional file 1, 2, respectively. As shown in Fig. 1B and 1C, the intranasal administration of GLY significantly increased cilia-driven flow compared with the control (PBS alone).

Ciliary motility was recorded using a microscope equipped with a high-speed digital camera (Additional file 3, 4). Kymographs of the movies under the conditions of GLY administration and the control are shown in Fig. 1D. An analysis of the ciliary beating revealed that CBF was significantly increased by the GLY administration compared with the control (Fig. 1E). The ciliary beating amplitude did not differ between the conditions (Fig. 1F). Both the effective stroke velocity and recovery stroke velocity were significantly greater with the GLY administration than with the control (Figs. 1G and H). The ratio of effective stroke velocity to recovery stroke velocity did not differ between the conditions (Fig. 1I).

Taken together, these results demonstrate that the 1-week continuous intranasal administration of GLY promotes the cilia-driven fluid flow and ciliary activity in murine airway epithelium.

### Short-term treatment with GLY or TIO promotes ciliary functions in the murine tracheal epithelium in organ culture models

**Fig. 2:**
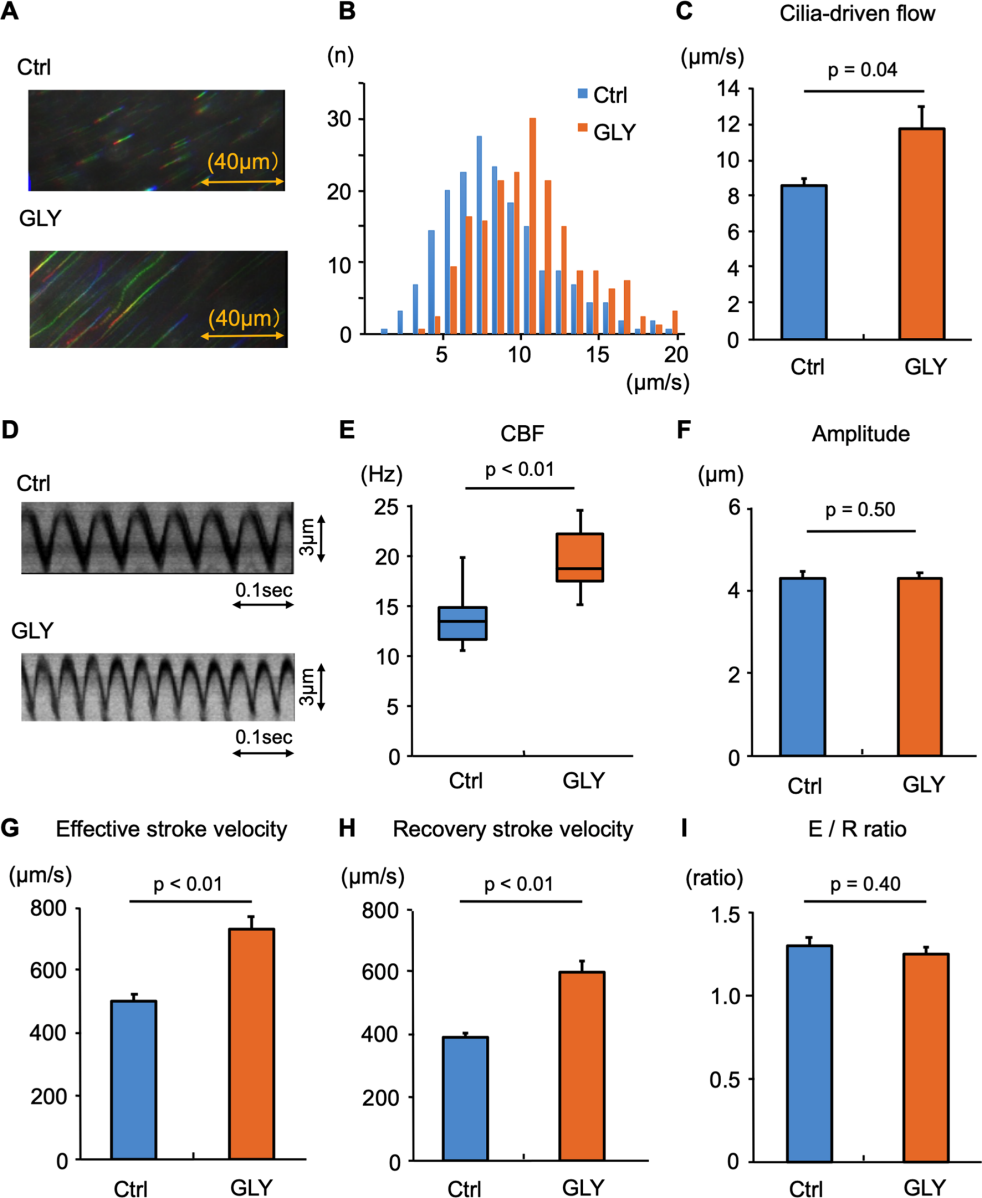
The short-term effects of glycopyrronium on cilia-driven flow and ciliary motility Murine tracheal epithelia were incubated for 1 h with/without glycopyrronium (GLY) in the culture medium. The cilia-driven flow and ciliary motility were then evaluated. **A.** Representative bead trajectories of cilia-driven flow (rainbow trace for 4.4 s). **B.** the histogram of the cilia-driven flows (200 beads from 4 tracheas) in each condition. **C.** the bar chart of the cilia-driven flow demonstrated that GLY significantly increased cilia-driven flow (control, 8.63 ± 0.39 μm/s; GLY, 11.85 ± 1.21 μm/s; n = 4 tracheas in each condition). **D.** Kymographs of ciliary beating. **E.** GLY significantly increased ciliary beat frequency (CBF; control, 13.48 [10.55–19.92] Hz; GLY, 18.75 [15.23–24.61] Hz; n = 30 cilia in each condition). **F.** The ciliary beating amplitude did not differ between the conditions (control, 4.31 ± 0.16 μm; GLY, 4.29 ± 0.15 μm). **G** and **H.** The effective stroke velocity (**G**) (control, 504.4 ± 20.7 μm/s; GLY, 734.9 ± 40.3 μm/s) and recovery stroke velocity (**H**) (control, 393.9 ± 15.1 μm/s; GLY, 602.2 ± 35.5 μm/s) were significantly increased by GLY treatment compared with the control. **I.** The ratio of effective stroke velocity to recovery stroke velocity (E/R ratio) did not differ between the two conditions (control, 1.30 ± 0.05; GLY, 1.25 ± 0.05). *Ctrl,* control

Continuous, i.e. long-term, administration of drugs might cause unknown changes in tracheal tissues. To rule out those uncertain indirect effects of LAMAs on ciliary functions in airway epithelium, we next investigated the effects of short-term treatment with GLY or TIO (1 h) in a tracheal organ culture model on cilia-driven flow and ciliary activity in murine tracheal epithelium. Figure 2A presents the representative bead trajectories with and without GLY treatment. As shown in Fig. 2B and C, GLY treatment for 1 h significantly increased cilia-driven flow compared with the control level. Figure 2D shows the kymographs of the movies in the presence or absence of GLY in the culture media. An analysis of the ciliary beating revealed that CBF was significantly increased by GLY treatment compared with the control findings (Fig. 2E). The ciliary beating amplitude did not differ between the conditions (Fig. 2F). Both the effective stroke velocity and recovery stroke velocity were significantly greater in the presence of GLY than in its absence (Fig. 2G and H). The ratio of effective stroke velocity to recovery stroke velocity did not differ between the conditions (Fig. 2I).

**Fig. 3:**
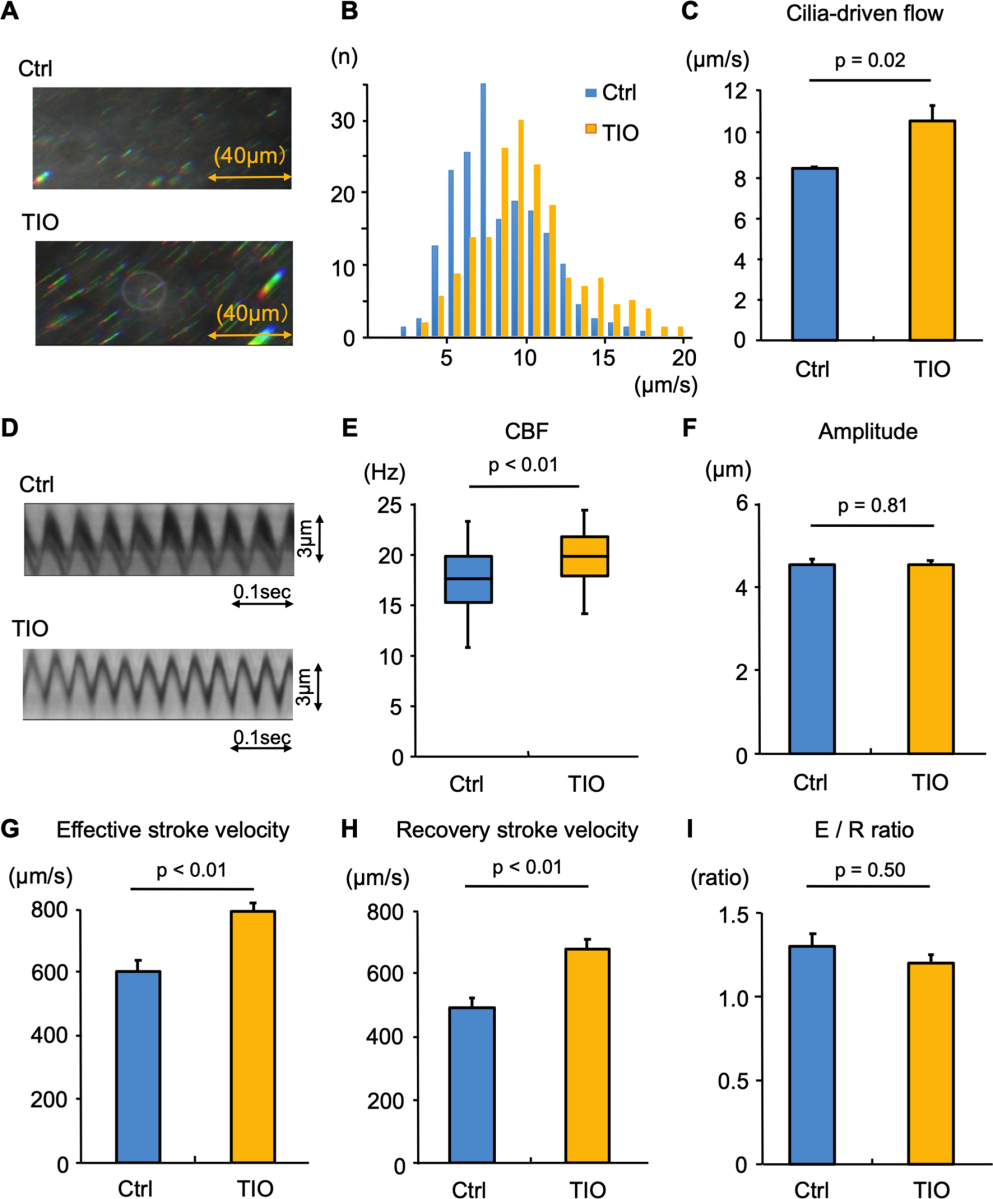
The short-term effects of tiotropium on cilia-driven flow and ciliary motility Murine tracheal epithelia were incubated for 1 h with/without tiotropium (TIO) in the culture medium. Cilia-driven flow and ciliary motility were then evaluated. **A.** Representative bead trajectories of cilia-driven flow (rainbow trace for 4.4 s). **B.** the histogram of the cilia-driven flows (200 beads from 4 tracheas) in each condition. **C.** the bar chart of the cilia-driven flow demonstrated that TIO significantly increased cilia-driven flow (control, 8.26 ± 0.11 μm/s; GLY, 10.59 ± 0.72 μm/s; n = 4 tracheas in each condition). **D.** Kymographs of ciliary beating. **E.** TIO significantly increased ciliary beat frequency (CBF; control, 17.58 [10.55–23.44] Hz; TIO, 19.92 [14.06–24.61] Hz; n = 30 cilia in each condition). **F.** The ciliary beating amplitude did not differ between the conditions (control, 4.56 ± 0.13 μm; TIO, 4.56 ± 0.10 μm). **G** and **H.** The effective stroke velocity (**G**) (control, 606.9 ± 33.3 μm/s; TIO, 794.0 ± 27.9 μm/s) and recovery stroke velocity (**H**) (control, 494.0 ± 30.7 μm/s; TIO, 683.1 ± 29.7 μm/s) were significantly increased by TIO treatment compared with the control. **I.** The ratio of effective stroke velocity to recovery stroke velocity (E/R ratio) did not differ between the two conditions (control, 1.29 ± 0.07; TIO, 1.20 ± 0.04). *Ctrl,* control

We performed the same experiments using TIO, another LAMA, to observe the promoting effects on cilia-driven flow and ciliary activity in murine tracheal epithelium. Figure 3A presents the representative bead trajectories. As shown in Fig. 3B and C, TIO treatment significantly increased cilia-driven flow compared with the control level. Figure 3D shows the kymographs of the movies in the presence or absence of TIO. An analysis of the ciliary beating revealed that CBF was significantly increased by TIO treatment compared with the control findings (Fig. 3E). The ciliary beating amplitude did not differ between the conditions (Fig. 3F). The effective stroke velocity and recovery stroke velocity were significantly greater in the presence of TIO than in the control (Fig. 3G and H). The ratio of effective stroke velocity to recovery stroke velocity did not differ between the conditions (Fig. 3I).

Collectively, these results indicate that short-term treatment with GLY or TIO have a potential to enhance cilia-driven flow and ciliary activity in murine airway epithelium.

### LAMAs directly increase CBF in NHBE cells cultured on ALI condition

**Fig. 4:**
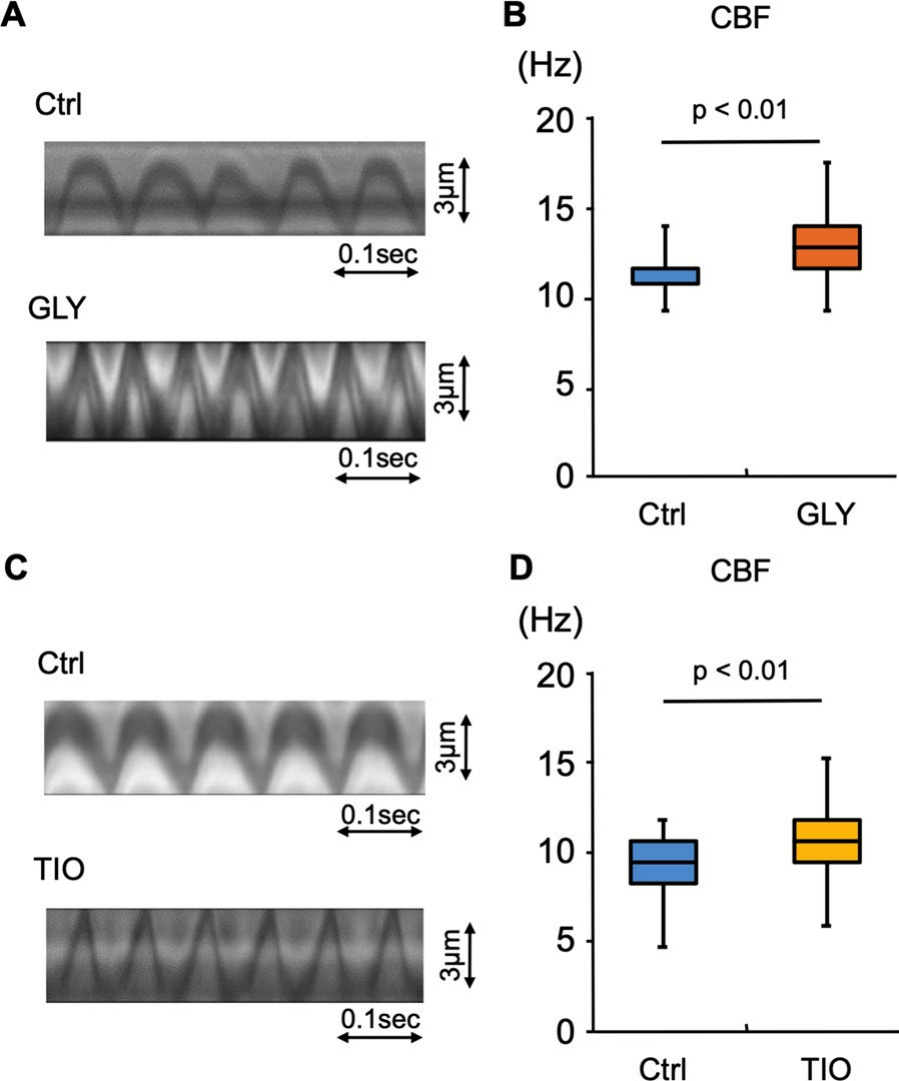
The effects of glycopyrronium or tiotropium on ciliary motility in normal human bronchial epithelial cells Well-differentiated normal human bronchial epithelial (NHBE) cells cultured under air–liquid interface (ALI) conditions for 4 weeks were treated for 1 h with/without glycopyrronium (GLY) or tiotropium (TIO) in the culture medium. Ciliary beat frequency (CBF) was then evaluated. **A.** Kymographs of ciliary beating. **B**. GLY significantly increased CBF (CBF; control, 11.72 [9.38–14.06] Hz; GLY, 12.89 [9.38–17.58] Hz; n = 30 cilia in each condition). **C.** Kymographs of ciliary beating. D. TIO significantly increased CBF (control, 9.38 [4.69–11.72] Hz; TIO, 10.55 [5.86–15.23] Hz; n = 30 cilia in each condition). *Ctrl,* control

We further aimed to determine whether LAMAs have a direct promoting effect on airway ciliary functions on the human airway epithelium. To facilitate ciliated cell differentiation, NHBE cells were cultured for 4 weeks under ALI condition. Using well-differentiated NHBE cells cultured under ALI conditions for 4 weeks, we investigated whether short-term (1 h) treatments with GLY or TIO increased CBF. Figure 4A and C shows the ciliary motion kymographs. As shown in Fig. 4B, CBF was significantly increased by GLY treatment for 1 h compared with the control. Similarly, TIO treatment significantly increased CBF in NHBE cells under ALI conditions (Fig. 4D).

Taken together, all the results from the experiments using mouse models and well-differentiated NHBE cell cultures provide evidence that LAMAs directly enhance ciliary activity and promote ciliary clearance in the airway epithelium.

### Search for the mechanism of LAMA-mediated promotion of ciliary functions

**Fig. 5:**
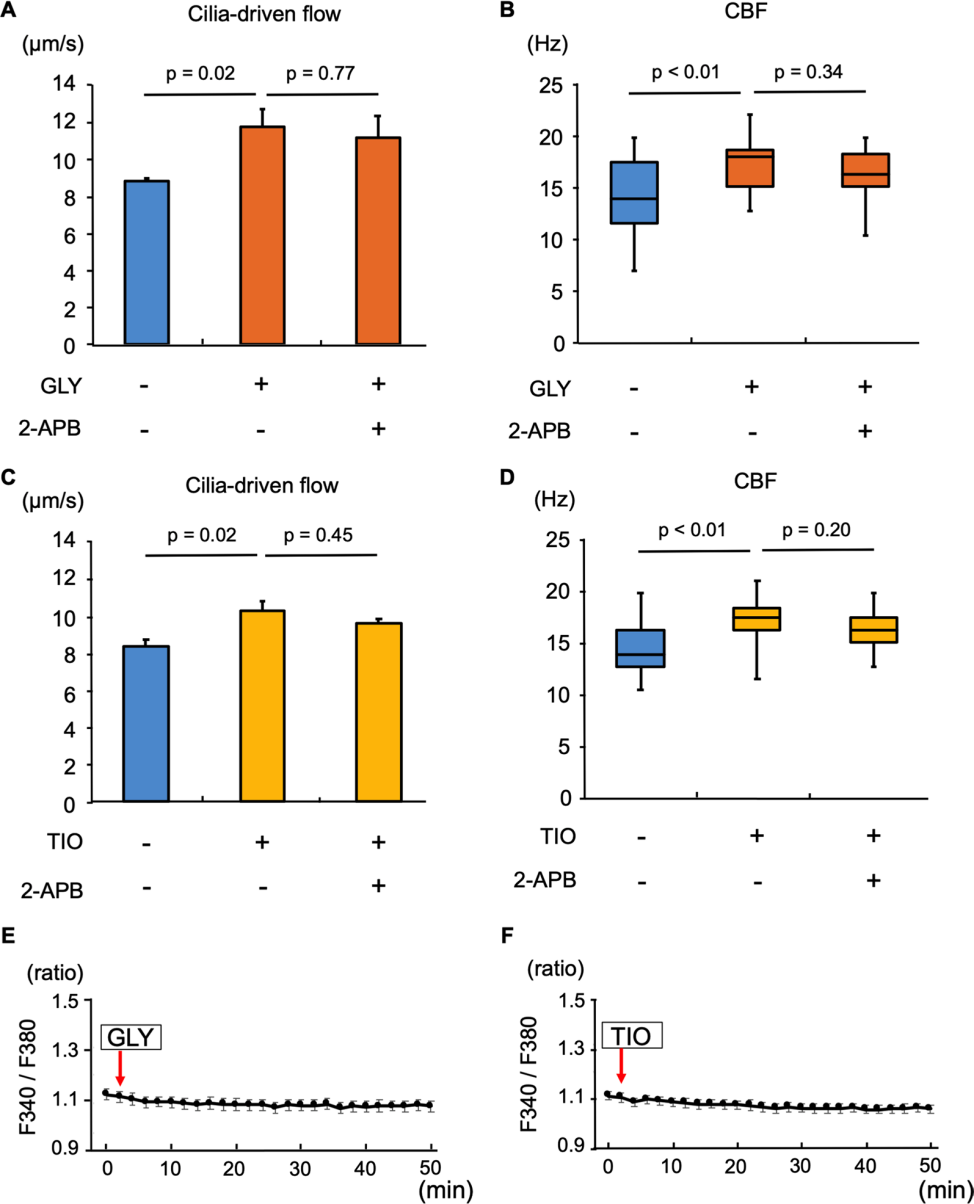
Involvement of intracellular calcium ion in the glycopyrronium or tiotropium-mediated promotion of ciliary function The murine tracheal tissues were incubated with glycopyrronium (GLY) or tiotropium (TIO) with/without 2-Aminoethoxydiphenylborane (2-APB) for 1 h. Cilia-driven flow and ciliary beat frequency (CBF) were then evaluated. **A** and **B**. 2-APB had few effects on GLY-mediated increases of cilia-driven flow (**A**) (control, 8.73 ± 0.15 μm/s; GLY, 11.66 ± 0.64 μm/s; GLY + 2-APB, 11.04 ± 0.84 μm/s; n = 4 tracheas in each condition) and CBF (**B**) (control, 14.06 [7.03–19.92] Hz; GLY, 18.17 [12.89–22.27] Hz; GLY + 2-APB, 16.40 [10.55–19.92] Hz; n = 30 cilia in each condition). **C** and **D**. 2-APB had few effects on TIO-mediated increases of cilia-driven flow (**C**) (control, 8.46 ± 0.34 μm/s; TIO, 10.37 ± 0.48 μm/s; TIO + 2-APB, 9.54 ± 0.38 μm/s; n = 3 tracheas in each condition) and CBF (**D**) (control, 14.06 [10.55–19.92] Hz; TIO, 17.58 [11.72–21.09] Hz; TIO + 2-APB, 16.40 [12.89–19.92] Hz; n = 30 cilia in each condition). **E** and **F**. Intracellular Ca^2+^ concentrations were measured using Fura-2/AM in normal human bronchial epithelial cells. Neither treatment with GLY (**E**) nor TIO (**F**) changed the fluorescence ratio (F340/F380) of Fura-2 until 50 min (n = 20 cells). *Ctrl,* control

We investigated the underlying mechanisms of LAMA-mediated promotion of ciliary functions. The change of intracellular Ca^2+^ concentration is one of the mechanisms that modulate ciliary beating in airway epithelium [28, 29]. To assess whether the increase of intracellular Ca^2+^ concentration was involved in the LAMA-mediated promotion of ciliary functions, we used 2-APB, an inhibitor of inositol-trisphosphate receptor, to inhibit Ca^2+^ release from endoplasmic reticula [30]. The murine tracheal tissues were incubated with GLY or TIO with or without 2-APB for 1 h, and cilia-driven flow and CBF were evaluated. The 2-APB showed no inhibitory effects on GLY-mediated increases in cilia-driven flow (Fig. 5A) and CBF (Fig. 5B). Similarly, the 2-APB had no effect on TIO-mediated increases in cilia-driven flow (Fig. 5C) and CBF (Fig. 5D).

We also tested whether LAMAs treatment affected intracellular Ca^2+^ concentrations in NHBE cells using Fura-2/AM. As shown in Fig. 5E and F, neither treatment with GLY nor TIO changed the fluorescence ratio (F340/F380) of Fura-2 until 50 min, suggesting that LAMAs have no effect on intracellular Ca^2+^ concentration. We confirmed that Fura-2 properly worked as an indicator of intracellular Ca^2+^ concentration in NHBE cells; TG increased Fura-2 fluorescence in NHBE cells (Additional file 5: Fig. S1A). In addition, subsequent TG treatment after 40 min treatment with GLY or TIO increased Fura-2 fluorescence in NHBE cells (Additional file 5: Figs. S1B, S1C), indicating that the NHBE cells were alive and responded to the drug till the end of the experiments.

**Fig. 6:**
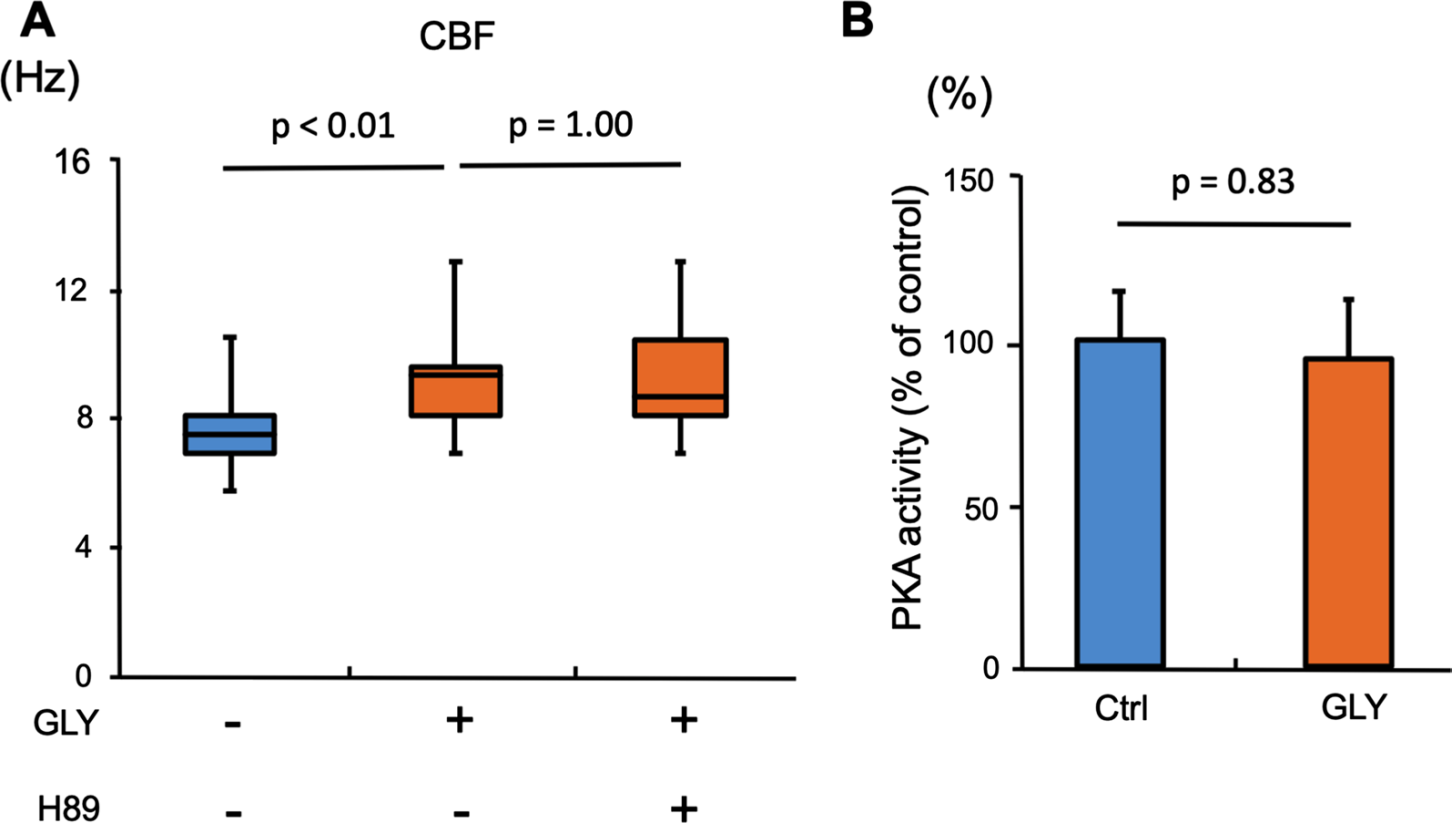
Examination of involvement of protein kinase A in the glycopyrronium-mediated increase of ciliary beat frequency Well-differentiated normal human bronchial epithelial (NHBE) cells were incubated with glycopyrronium (GLY) with or without H89, a protein kinase inhibitor with a high specificity for protein kinase A (PKA), for 1 h. Ciliary beat frequency (CBF) and PKA activity was then evaluated. **A.** H89 had few effects on GLY-mediated increases in CBF (control, 7.62 [5.86–10.55] Hz; GLY, 9.38 [7.03–12.89] Hz; GLY + H89, 8.79 [7.03–12.89] Hz; n = 30 cilia in each condition). **B.** GLY treatment had few effects on PKA activity (control, 100 ± 14.95%; GLY, 94.53 ± 18.14%; n = 3). The results are presented as percent of control. *Ctrl,* control

A former study reported that PKA activation by isoproterenol increased CBF in bovine bronchial epithelial cells [31]. We thus assessed whether cAMP-dependent PKA activation was involved in GLY-mediated increases in CBF. Well-differentiated NHBE cells cultured under ALI conditions for 4 weeks were treated with GLY with or without H89, a protein kinase inhibitor with a high specificity for PKA [28], for 1 h; CBF was then evaluated. The addition of H89 had no effect on the GLY-mediated increase in CBF (Fig. 6A). GLY treatment also had no effect on PKA activity (Fig. 6B), suggesting that PKA pathways are not involved in GLY-mediated increases in CBF.

Extracellular ATP is also known to increase CBF and hasten MCC in the airways by activating purinergic P2 receptor (P2R) signaling [10, 32]. Therefore, we next evaluated whether GLY induces release of ATP to extracellular milieu from murine tracheal tissues. ATP concentrations were measured in the culture supernatants with or without GLY treatment for 5–120 min. As shown in Additional file 5: Fig. S2, ATP concentrations did not differ between the GLY treatment and the control, indicating that GLY had no effect on extracellular ATP release.

### Antagonists of M3 receptor, but neither M1 nor M2 receptor, have the potential to promote ciliary functions in the murine tracheal epithelium

Bronchial epithelial cells express M1 and M2 receptors, as well as M3 receptor [21, 33]. Therefore, we investigated the action of M1 and M2 receptor antagonists, and M3 receptor antagonist other than LAMAs on airway ciliary function using telenzepine (M1 receptor antagonist), gallamine (M2 receptor antagonist), and 4-DAMP (4-Diphenylacetoxy-N-methylpiperidine methiodide, M3 receptor antagonist). We have assessed the effects of treatment with telenzepine, gallamine, or 4-DAMP (1 h) in a tracheal organ culture model on cilia-driven flow and ciliary activity in murine tracheal epithelium. As shown in Additional file 5: Fig. S3A, 4-DAMP treatment significantly increased cilia-driven flow compared with the control level, whereas telenzepine and gallamine did not. An analysis of the ciliary beating revealed that CBF was significantly increased by 4-DAMP treatment compared with the control findings, but treatment with telenzepine and gallamine had no effect on CBF (Additional file 5: Fig. S3B). These results suggest that M3 receptor antagonists, including GLY and TIO, but neither M1 nor M2 antagonists, have the potential to promote ciliary function in airway epithelium.

## Discussion

This study describes the novel effects of LAMAs on ciliary functions, with particular focusing on ciliary activity and cilia-driven flow, in airway epithelium. Our findings revealed that the intranasal administration of GLY increases ciliary activity and cilia-driven flow in murine airway epithelium. Using an organ culture of murine tracheal tissues and a primary culture of well-differentiated NHBE, we confirmed that both GLY and TIO directly increased CBF and cilia-driven flow in airway epithelium. Together, these results show that LAMAs, which are major bronchodilators for treating asthma and COPD, have the potential to enhance airway ciliary function and improve MCC.

The present study clearly indicated that LAMAs are multifunctional agents that not only relax airway smooth muscle as bronchodilators but also to enhance ciliary functions in airway epithelium, which provides effective MCC in the airway. We first conducted in-vivo experiments using the intranasal administration of GLY in mice, which represents an inhalation administration of LAMAs in the real clinical setting of asthma and COPD. The continuous intranasal administration of GLY significantly increased cilia-driven flow and ciliary activity; however, it remains unclear whether these are the direct effects of GLY on airway epithelial cells or indirect effects via diverse cellular and humoral factors. We therefore conducted in-vitro experiments using a murine tracheal organ culture to investigate the short-term effects of two types of LAMAs (GLY and TIO), on airway epithelium, and demonstrated that both GLY and TIO directly enhance ciliary functions in murine airway epithelium. Additionally, we further validated the LAMA-mediated promotion of airway ciliary function using human bronchial epithelial cells. In consistent with our findings, Yaghi et al*.* formerly reported that treatment with TIO increased CBF in nasal ciliated cells taken from COPD patients [18]. Taken together, inhaled LAMAs in clinical practice might provide not only bronchodilation but also promotion of airway ciliary function, which relieves the clinical symptoms of airflow obstruction, and improves MCC and susceptibility to respiratory tract infections in patients with asthma and COPD.

In the present study, we clarified direct actions of LAMAs on promoting airway ciliary function using primary culture of well-differentiated NHBE cells. Primary culture of NHBE cells have the differentiation potential into ciliated cells under ALI culture conditions [25, 26]. Therefore, this culture system enabled us to evaluate ciliary motion of ciliated cells without considering other cellular components. Using the well-differentiated NHBE cells under ALI conditions, we were able to evaluate ciliary motion by the imaging techniques and confirmed that LAMAs directly increased CBF in human airway epithelial cells.

Several studies have demonstrated that LAMAs exerted a variety of additive effects, including off-target effects [34–38], although the primary function of LAMAs is bronchodilation to block M3 receptor activity and relax smooth muscle in the airways. In the in vivo model, LAMAs showed anti-inflammatory activity, such as a decrease in cytokine levels in the bronchoalveolar lavage and inhibition of neutrophil chemotactic activity and eosinophil recruitment [34, 35], as well as inhibitory effects in airway remodeling [34]. In terms of LAMA actions on airway epithelial cells, Kistemaker et al*.* demonstrated that TIO inhibited IL-13-induced goblet cell metaplasia in human airway epithelial cells [37], which might be useful for protecting against mucus hypersecretion especially in patients with asthma. This study’s findings elucidated the novel direct actions of LAMAs on airway epithelial cells: the promotion of airway ciliary functions. We also confirmed this promoting effect of LAMAs on airway ciliary function using two different types of LAMAs, GLY and TIO. Under the disease conditions with excessive acetylcholine (ACh) [38–40] (e.g. asthma and COPD), LAMAs exert multiple therapeutic effects, including bronchodilation, anti-inflammatory activity, protection of mucus hypersecretion, and promoting ciliary functions.

Although our findings reveal the potential of LAMAs to increase cilia-driven flow and ciliary activity in airway epithelium, the intracellular mechanisms of these stimuli are not fully understood. So far, a variety of mechanisms (e.g., intracellular Ca^2+^ concentration, PKA activation, and ATP-P2R pathway) have been reported to be involved in the regulation of CBF and ciliary activities in airway epithelium [10, 28, 31, 32, 41, 42]. In this study, we conducted several experiments to elucidate the underlying mechanism of LAMA-mediated promotion of ciliary function; however, we could not identify the specific mechanisms in these effects. Cassambai et al. recently showed that TIO increased intracellular Ca^2+^ concentration in cardiomyocytes, resulting in cell damage within the heart [43]. We investigated whether LAMAs treatment affected intracellular Ca^2+^ concentrations in NHBE cells using Fura-2/AM in this study. In contrast to their findings, we observed no increase in Ca^2+^ concentration in the NHBE cells after the TIO or GLY treatment. The difference in cell types might result in different LAMA actions.

A few studies reported that cholinergic agonists (e.g. ACh) also increased CBF and MCC via activation of muscarinic receptors [44–46]. In the present study, we have shown that M3 receptor antagonists, including GLY and TIO, but not M1 or M2 antagonists, have a specific potential to promote airway ciliary functions (Additional file 5: Fig. S3), although the precise mechanisms of LAMA-mediated increase of ciliary function were not fully understood. On the contrary, Klein et al*.* demonstrated that M3 receptor stimulation by muscarine increased cilia-driven particle transport in the murine airway [44]. The mechanisms underlying these conflicting findings that both agonists and antagonists of the M3 receptor have similar effects on airway ciliary function remain to be elucidated and further studies are warranted.

LAMAs might have unique mechanisms of action to stimulate ciliary function in airway epithelium. Interestingly, Birrell et al*.* reported the off-target effects of TIO in airway sensory nerves, in which inhaled TIO blocked capsaicin-induced coughing to inhibit the neuronal transient receptor potential V1, which was unrelated to anticholinergic activity [36]. Similar off-target effects for LAMAs might be involved in the promotion of ciliary function in the airway epithelium. Elucidating the underlying mechanisms of the LAMA-mediated promotion of airway ciliary function will be an important topic for exploration in future studies.

## Conclusions

This study provides evidence that LAMAs, which are major bronchodilators for treating asthma and COPD, promote cilia-driven flow and ciliary activity in mouse and human airway epithelium. This study’s findings imply that this direct LAMA action on airway epithelium might enhance MCC, in combination with its protective effects on airflow restriction, which prevents susceptibility to respiratory tract infections and exacerbation in patients with asthma and COPD. Further investigations are warranted to elucidate the underlying mechanisms of the direct effects of LAMAs on the promotion of airway ciliary function.

## Supplementary Information

**Additional file1**. Cilia-driven flow over the murine tracheal surface using small polystyrene beads under the condition treated with PBS intranasally for 7 days.

**Additional file2**. Cilia-driven flow under the condition treated with GLY intranasally for 7 days.

**Additional file3**. Ciliary motility under the condition treated with PBS intranasally for 7 days.

**Additional file4**. Ciliary motility under the condition treated with GLY intranasally for 7 days.

**Additional file5. Figure S1**: Changes of intracellular calcium ion concentration by thapsigargin treatment. **Figure S2**: ATP concentration in the culture supernatant. **Figure S3**: The action of M1, M2, and M3 receptor antagonists on airway ciliary function.

## Data Availability

The datasets used and/or analyzed during the current study are available from the corresponding author on reasonable request.

## Abbreviations

ACh: Acetylcholine
ALI: Air–liquid interface
ATP: Adenosine triphosphate
CBF: Ciliary beat frequency
COPD: Chronic obstructive pulmonary disease
GLY: Glycopyrronium
H89: N-[2-(p-bromocinnamylamino) ethyl]-5-isoquinolinesulfonamide, dihydrochloride
ICS: Inhaled corticosteroids
LABAs: Long-acting β2-agonists
LAMAs: Long-acting muscarinic antagonists
MCC: Mucociliary clearance
NHBE: Normal human bronchial epithelial
PBS: Phosphate-buffered saline
PKA: Protein kinase A
P2R: P2 receptors
TG: Thapsigargin
TIO: Tiotropium
WT: Wild-type
2-APB: 2-Aminoethoxydiphenylborane
4-DAMP: 4-Diphenylacetoxy-N-methylpiperidine methiodide
