# Supplementary material for: Effects of long-acting muscarinic antagonists on promoting ciliary function in airway epithelium
Source: BMC Pulm Med. 2022 May 8;22:186. doi: 10.1186/s12890-022-01983-3 (PMC9080152; doi:10.1186/s12890-022-01983-3)
Supplement: Supplementary file 5 — Additional file5. Figure S1: Changes of intracellular calcium ion concentration by thapsigargin treatment. Figure S2: ATP concentration in the culture supernatant. Figure S3: The action of M1, M2, and M3 receptor antagonists on airway ciliary function. [file 12890_2022_1983_MOESM5_ESM.docx]

**Supplementary Figures**

**Figure S1.**


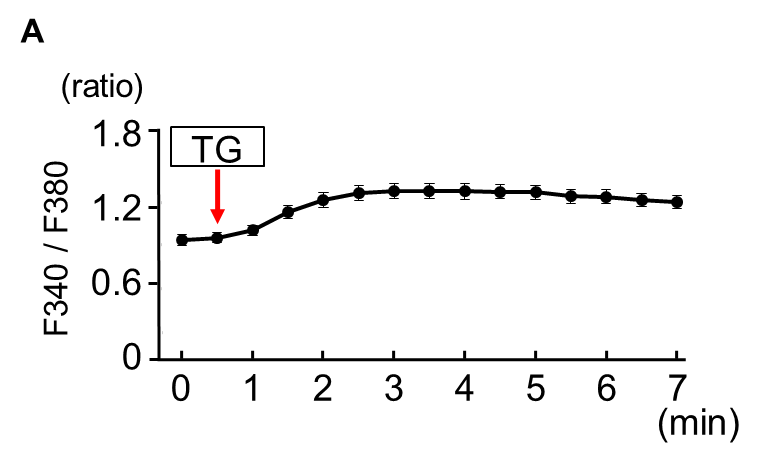


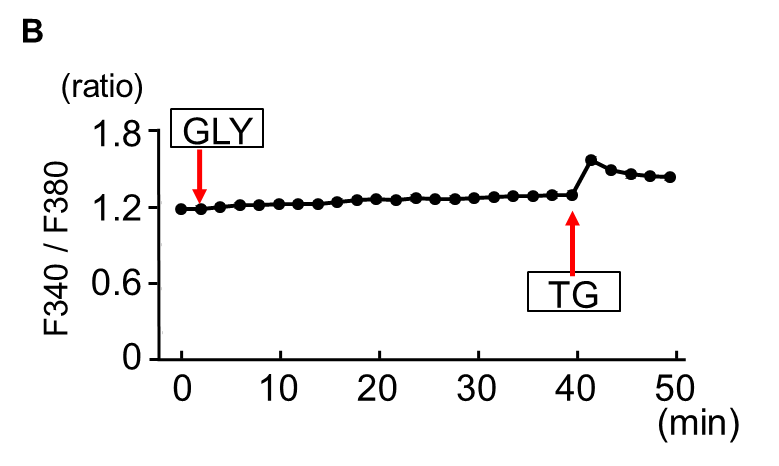


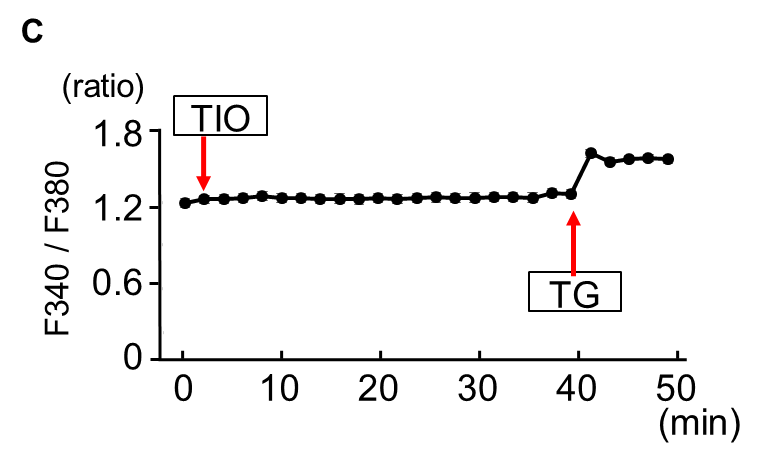


**Figure S1. Changes of intracellular calcium ion concentration by thapsigargin treatment**

Intracellular calcium ion concentration was measured using fura-2/AM in normal human bronchial epithelial (NHBE) cells. **A.** Thapsigargin (TG) increased Fura-2 fluorescence in NHBE cells (n = 20 cells). **B.** TG increased Fura-2 fluorescence in NHBE cells after 40 min treatment with glycopyrronium (GLY) (n = 20 cells). **C.** TG increased Fura-2 fluorescence in NHBE cells after 40 min treatment with tiotropium (TIO) (n = 20 cells).

**Figure S2.**


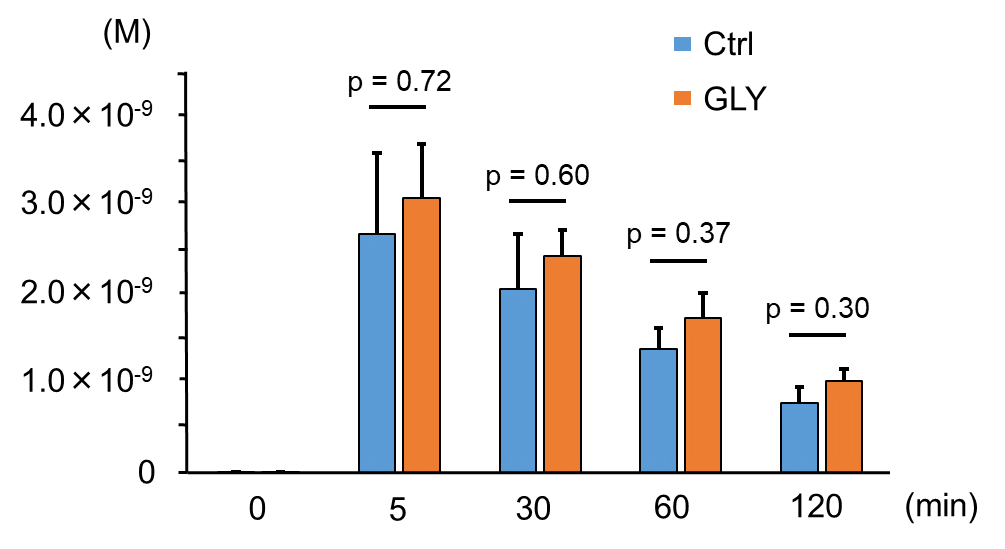


**Figure S2. ATP concentration in the culture supernatant**

Using the murine tracheal tissue culture system, ATP concentrations were measured in the culture supernatants with or without glycopyrronium (GLY) treatment for 5–120 min. ATP concentrations did not differ between the GLY treatment and the control (n = 5 each). *Ctrl,* control.


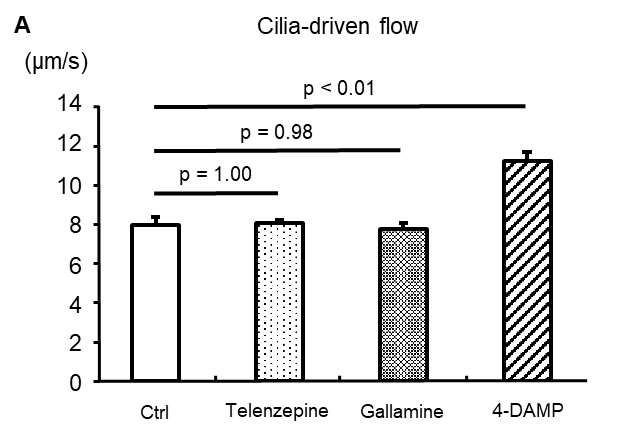
**Figure S3.**


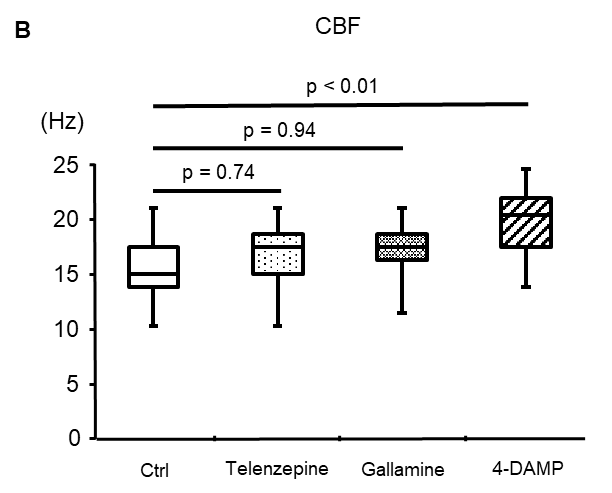


**Figure S3. The action of M1, M2, and M3 receptor antagonists on airway ciliary function**

The murine tracheal tissues were incubated with telenzepine (M1 receptor antagonist), gallamine (M2 receptor antagonist), or 4-DAMP (4-Diphenylacetoxy-N-methylpiperidine methiodide, M3 receptor antagonist) for 1 h. **A** and **B**. Only 4-DAMP increased cilia-driven flow (A) (control, 8.00 ± 0.43 μm/s; Telenzepine, 8.10 ± 0.20 μm/s; Gallamine, 7.79 ± 0.33 μm/s; 4-DAMP, 11.29 ± 0.49μm/s; n = 3 tracheas in each condition) and ciliary beat frequency (CBF) (B) (control, 15.23 [10.55–21.09] Hz; Telenzepine, 17.58 [10.55–21.09] Hz; Gallamine, 17.58 [11.72–21.09] Hz; 4-DAMP, 20.51 [14.06–24.61] Hz; 10 cilia in each trachea were analyzed and experiments were repeated three times. In total, n = 30 cilia in each condition). *Ctrl,* control.
